# Giant right coronary artery aneurysm- case report and literature review

**DOI:** 10.1186/1749-8090-4-18

**Published:** 2009-05-01

**Authors:** Neerod K Jha, Husam Z Ouda, Javed A Khan, Gregory P Eising, Norbert Augustin

**Affiliations:** 1 Department of Adult Cardiac Surgery, Sheikh Khalifa Medical City (Managed by Cleveland Clinic), Abu Dhabi, UAE; 2 Division of Cardiovascular Medicine, Tawam-Johns Hopkins Hospital, Al Ain, UAE

## Introduction

Although the coronary artery aneurysms (CAA) are not uncommon, giant aneurysms are rare. Clinical presentation, prognosis and management of giant CAA are not well defined due to limited experience. Recently, there are increasing reports suggesting the occurrence of CAA as a complication of drug eluting stent implantation and angioplasty. Therefore, there is a need to report present case and review the available literature to remind, update and discuss this anomaly for better awareness, understanding and management especially in view of the expected increase in their incidence.

## Case presentation

A 54 years-old-hypertensive male patient was presented to us with history of recent inferior wall myocardial infarction (MI) which was managed with medical treatment in the referring hospital. On clinical examination, there was a 3/6 ejection systolic murmur along the left lower sternal border. Electrocardiogram was consistent with inferior MI. The 2-D echocardiogram revealed a large cystic mass adjacent to the right atrium. Coronary angiography revealed significant coronary artery disease in the proximal left anterior descending artery (LAD) and a giant aneurysm of middle segment of right coronary artery (RCA). There was a mild ectatic segment in the proximal circumflex coronary artery, as well.

Patient underwent successful resection of giant aneurysm of RCA under cardiopulmonary bypass (CPB) via median sternotomy. Proximal and distal communications of RCA were ligated from within the aneurysmal sac and then coronary artery bypass graft surgery (CABG) was performed using right internal mammary artery to the distal RCA and left internal mammary artery to the LAD.

**Figure 1:**
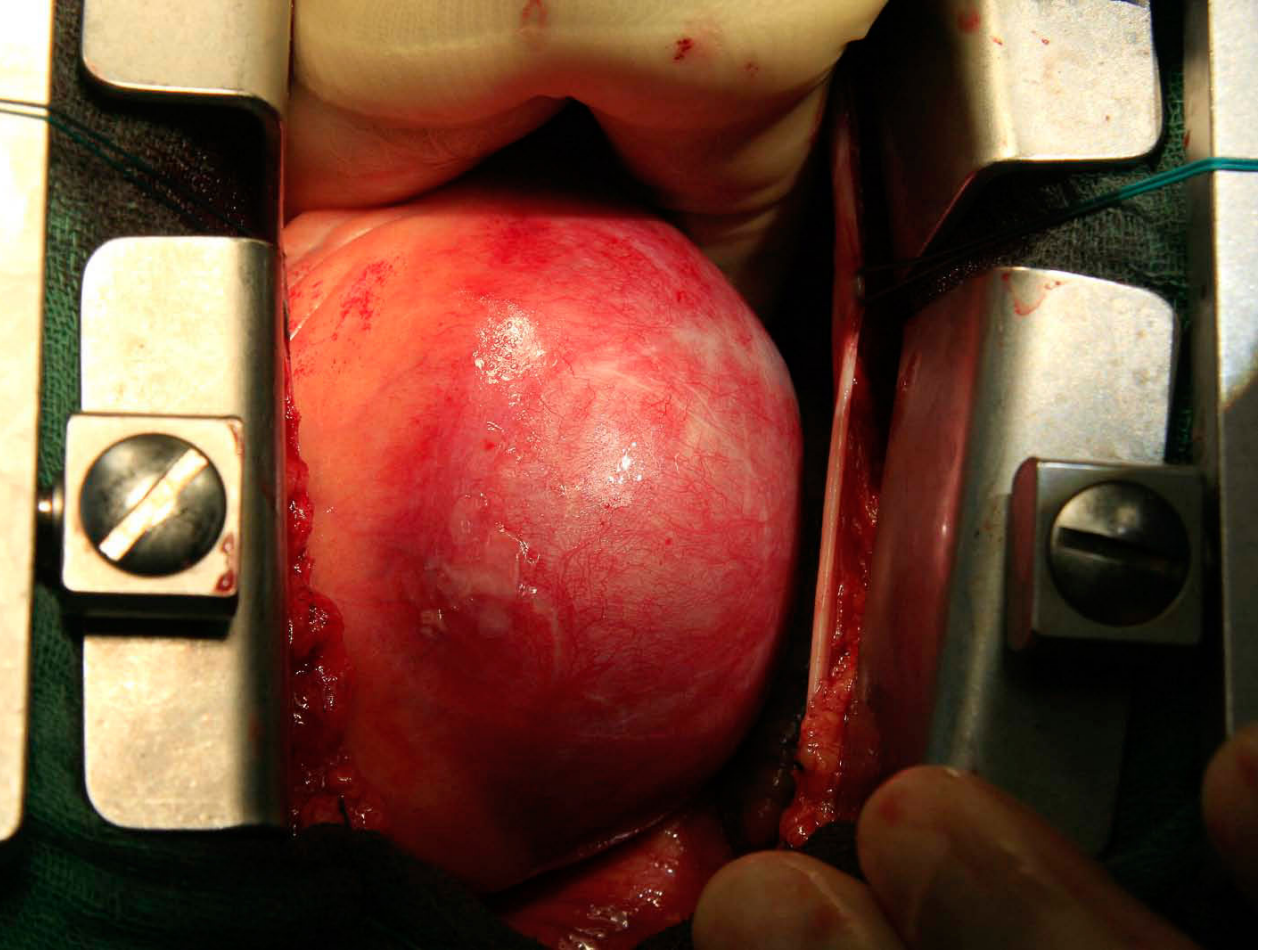
**Operative photograph showing a giant aneurysm of the right coronary artery**.

**Figure 2:**
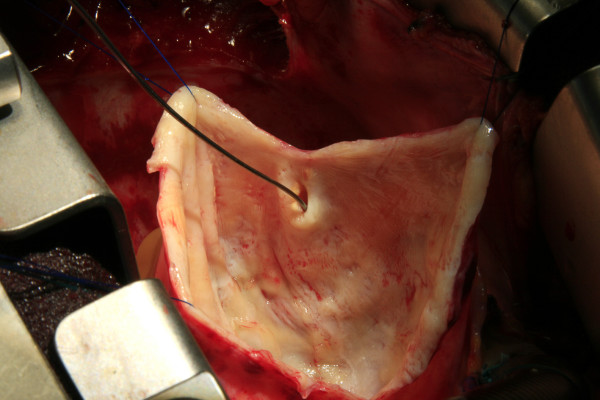
**Operative photograph showing inside view of the aneurysmal sac**. The tip of probe is within the proximal communication of right coronary artery.

The aneurysmal sac was found to be 12 × 9 × 1 cms in dimension, occupying the entire right atrioventricular groove and displacing the right atrium (Figure 1 &2). There was no luminal thrombus or calcification. Histopathology of excised aneurysm had shown widespread myxoid degeneration in the media, focal necrosis, atherosclerosis and fibrosis of the medial muscles.

## Discussion

Coronary artery aneurysm is defined as a localized dilatation exceeding the diameter of adjacent normal segment by 50% and occurs in approximately 1.5–5% of patient undergoing coronary angiography [1]. However, a coronary artery with a diameter more than 2 cm is termed as "giant aneurysm " and only a few cases have been described in the literature [2-23] (Table 1).

**Table 1:** Reported cases of giant coronary artery aneurysm in adults

**Author**	**Year **	**Number** **(patients)**	**Site**	**Presentation **	**Etiology **	**Management**
**Nobrega**[2]**'et al'**	1996	1	RCA, LAD	MI	SLE	Medical

**Channon 'et al'**[3]	1998	1	RCA	Medistinal mass	Atherosclerotic	Medical

**Yu 'etal'**[4]	2001	2	RCA, LAD	LV fistula	Congenital	Surgical

**Konen 'et al'**[5]	2001	1	RCA	Medistinal mass	Atherosclerotic	Surgical

**Hao 'et al'**[6]	2003	1	RCA	Angina	Atherosclerotic	Surgical

**Banerjee 'et al'**[7]	2004	1	RCA	Medistinal mass	Atherosclerotic	Medical

**Grandmougin 'et al'**[8]	2005	1	RCA	Cardiac tumour	Atherosclerotic	Surgical

**McGlinchey 'et al'**[9]	2005	1	RCA	Cardiac compression	Atherosclerotic	Surgical

**Dianyuan 'et al'**[10]	2005	6	3RCA,2LAD,1Diagonal	LV fistula	Congenital, Atherosclerotic	Surgical

**Shakir 'et al'**[11]	2005	1	RCA, LAD	CHF	Hypercholesterolemia	Surgical

**Augustin 'et al'**[12]	2006	1	RCA	Cardiac compression	Atherosclerotic	Surgical

**Kumar 'et al' **[13]	2006	1	RCA	SVC syndrome	Atherosclerotic	Surgical

**Manghat 'et al'**[14]	2006	1	LAD	Chest pain	Kawasaki disease	Medical

**Jindal 'et al'**[15]	2007	1	LAD	Angina	Stent implantation	Surgical

**Takano 'et al'**[16]	2007	1	RCA	MI	Atherosclerotic	Surgical

**Blank 'et al'**[17]	2007	1	RCA	Medistinal mass	Atherosclerotic	Surgical

**Malero 'et al'**[18]	2008	1	RCA	Intracardiac mass	Atherosclerotic	Surgical

**Vlachou 'et al'**[19]	2008	1	RCA	Medistinal mass, MI	Atherosclerotic	Surgical

**Eshtehardi'et al'**[20]	2008	1	LAD	Angina	Atherosclerotic	Stenting

**Matsubayashi 'et al'**[21]	2008	1	LM	Angina	Atherosclerotic	Surgical

**Kanaan 'et al'**[22]	2008	1	RCA, LAD	Angina	Takayasu disease	Surgical

**Sharma 'et al'**[23]	2009	1	LAD	Angina	Stent implantation	Medical

RCA-right coronary artery, LAD-left anterior descending artery, LM-left main coronary artery, MI-myocardial infarction, CHF-congestive heart failure, LV-left ventricle, SVC-superior vena cava, SLE-systemic lupus erythematosus

In adults, CAA is predominantly atherosclerotic in origin however, other causes include Kawasaki disease, autoimmune disease, trauma, infection, dissection, congenital malformation and angioplasty [1,24]. Recently, with the advent of implantation of drug eluting stents there are increasing reports suggesting stents causing coronary aneurysm months or years after the procedure [1,14,22,24]. The proposed pathogenesis of stent-related aneurysm formation is multi-factorial. The drug-eluting stent contains immunosuppressant such as Sirolimus, which inhibits inflammation, or chemotherapeutic agents like Paclitaxel, which is an anti-inflammatory agent and inhibits cell proliferation. In due course of time, once drug is eluted, the polymer in which the drug is embedded may elicit a hypersensitivity reaction and vasculitis and results in weakening of vessel wall and subsequent dilatation [1,14,22,24]. Mechanical damage to the arterial wall during balloon angioplasty and stent placement or turbulent blood flow may be an added factor for the development of an aneurysm [1].

The majority of the patients with CAA are asymptomatic but they may present with angina pectoris, myocardial infarction, sudden death or complications such as thrombus formation, embolisation, fistula formation, rupture, hemo-pericardium, tamponade, compression of surrounding structure or congestive heart failure [1,3,4,9-12]. Coronary artery aneurysms are small, thick-walled structures with a relative low-risk of rupture but usually associated with myocardial ischemia [5] However, giant CAA are associated with advance age, tendency for complications including rupture and may present as mediastinal, intracardiac mass or superior vena cava syndrome in addition to ischemic symptoms [3,5,7,13,17-19].

Clinical presentation of giant CAA may mimic aneurysm of ascending aorta, pulmonary trunk, cardiac tumour, pericardial tumour or thymoma [1]. Giant CAA may be detected by non-invasive tools like echocardiography, computed tomography, magnetic resonance imaging but coronary angiography remains the gold standard which provides information about size, shape, location and co-existing anomalies such as coronary artery disease [24]. Since, our patient presented with MI, a diagnostic coronary angiography was done straightaway.

Due to rarity and non-availability of controlled trials, there is no optimal management strategy for patients with giant CAA. However, depending on the symptoms, etiology and associated lesions medical treatment (anti-platelet agent, anti-coagulation), stent implantation or surgical exclusion of the aneurysm using resection or ligation technique have been described [1,10]. A review of literature suggests that surgery is the preferred approach for Giant CAA in order to achieve excision of the aneurysmal sac, manage associated surgical condition and prevent complications [2-23].

The surgical management requires median sternotomy, cardiopulmonary bypass and myocardial revascularization (CABG). Occasionally femoral artery is cannulated for CPB to decompress the aneurysm and the ventricle before opening the chest for safety [10]. Prognosis of CAA is controversial but overall 5-year survival is reported to be 71% [1,24].

Therefore, Giant CAA is an uncommon lesion with varied clinical presentation and should be considered in the differential diagnosis of other conditions mimicking similar symptoms and need proper use of imaging technology to diagnose this rare anomaly and associated lesions for successful management. Surgical management need to be planned carefully and requires appropriate techniques for a better outcome.

## Consent

Written informed consent was obtained from the patient for publication of this case report including pictures for review.

## Competing interests

The authors declare that they have no competing interests.

## Authors' contributions

NJ collected the data and has written the manuscript. HO is the referring and treating cardiologist. JK is co-author and assisted the surgery. NA is consultant in-charge surgeon who operated upon the patient. GE is also a senior author who also managed this case.
